# The Inter-Mammary Sticky Roll: A Novel Technique for Securing a Doppler Ultrasonic Probe to the Precordium for Venous Air Embolism Detection

**DOI:** 10.7759/cureus.719

**Published:** 2016-08-01

**Authors:** David R Santiago-Dieppa, Arvin R Wali, Brandon C Gabel, Alexander A Khalessi, Hoi Sang U, John C Drummond

**Affiliations:** 1 Department of Neurosurgery, University of California, San Diego; 2 Department of Anesthesiology, University of California, San Diego

**Keywords:** venous air embolism, precordial doppler, neurosurgery, neuroanesthesia, intermammary sulcus, intermammary cleft, inter-mammary sticky roll, adhesive tape, surgical towel, probe positioning

## Abstract

Venous air embolism is a devastating and potentially life-threatening complication that can occur during neurosurgical procedures. We report the development and use of the “inter-mammary sticky roll,” a technique to reliably secure a precordial Doppler ultrasonic probe to the chest wall during neurosurgical cases that require lateral decubitus positioning. We have found that this noninvasive technique is safe, and effectively facilitates a constant Doppler signal with no additional risk to the patient.

## Introduction

Venous air embolism (VAE) is defined as air that becomes entrained within venous structures [1]. Although a VAE can occur during any invasive intervention, the incidence during neurosurgical procedures, in particular those involving the posterior fossa, has been reported to be as high as 82.6% [2]. A precordial Doppler is the standard of care for monitoring situations in which significant risk of VAE is anticipated.

VAE that remains unrecognized or untreated can rapidly result in cardiovascular collapse and death. Thus, given the high potential for VAE in neurosurgery, tools and techniques that facilitate the early recognition and treatment have long been considered important in neurosurgical anesthesia.

While a variety of methods have been used for the intra-operative detection of VAE, the contemporary standard of care entails only the precordial Doppler and expired carbon dioxide analysis [3]. The precordial Doppler is among the oldest of methods employed for VAE detection [4]. It is entirely non-invasive and is associated with almost no risk of morbidity to the patient. However, the use of precordial Doppler is directly dependent on the ultrasonic probe’s intimate and stable apposition to the chest wall. These requirements occasionally, in particular in the presence of obesity or generous breast tissue, make its use in the lateral position exceedingly difficult. We have developed a technique that increases the probe’s contact and pressure against the precordium and results in a more reliable and continuous Doppler signal.

## Technical report

**Figure 1:**
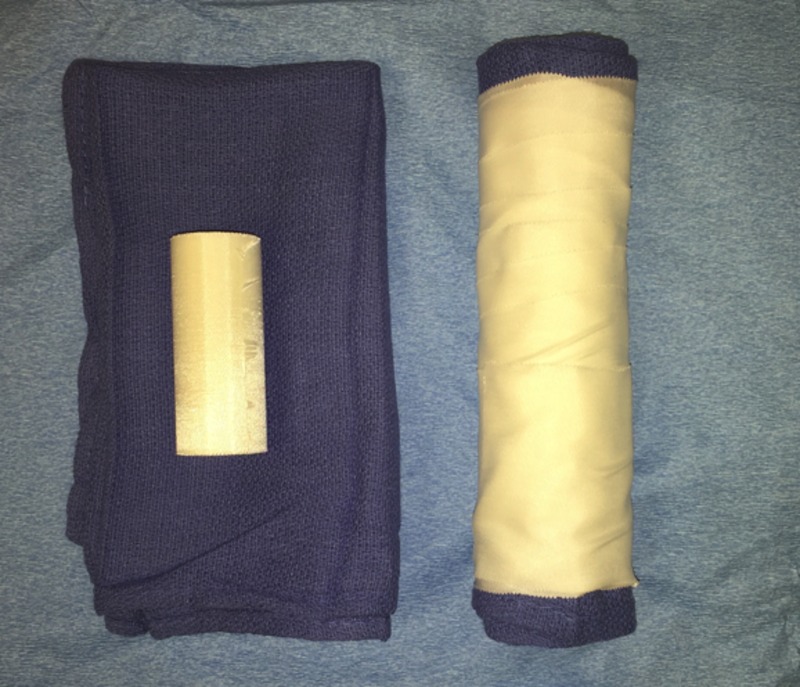
Left: Supplies Used for Fabrication of the IMSR; Right: Completed IMSR

Fabrication of the inter-mammary sticky roll (IMSR) is exceedingly simple and inexpensive. The IMSR is a smaller version of the so-called “sticky rolls" used in some operating rooms to maintain the thorax in the lateral position. For the IMSR, a 60 x 40 cm operating room surgical towel is folded in half along the long axis. The leading 20 cm edge of the towel is then rolled to completion. Three-inch medical grade adhesive tape is then wrapped circumferentially around the towel with the adhesive side of the tape facing outward (Figure 1).

**Figure 2:**
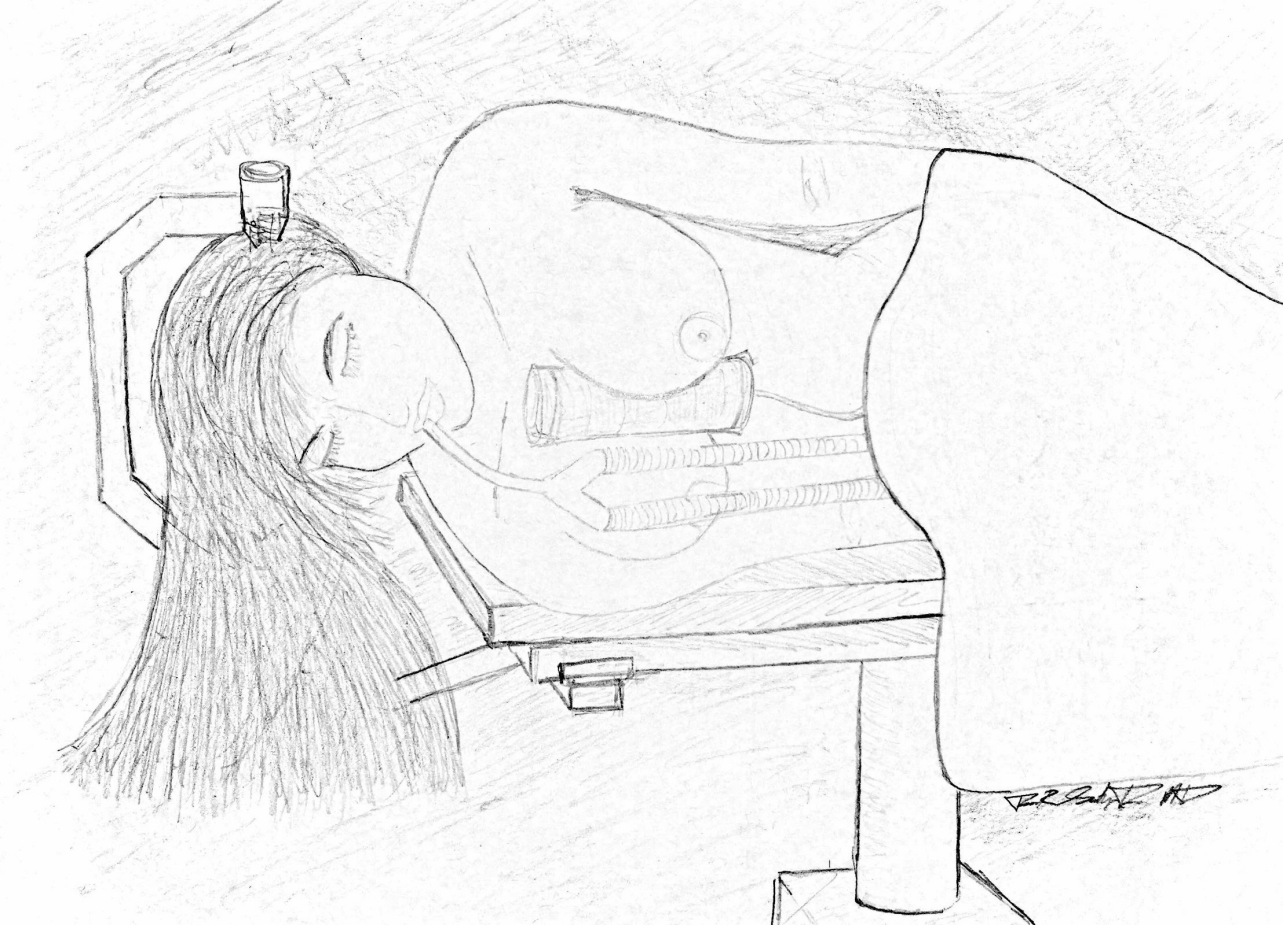
Artist's Rendering of a Patient in the Lateral Decubitus Position with Proper IMSR Placement in the Intermammary Sulcus.

Once the patient has been placed in the lateral decubitus position, the precordial Doppler probe is manually applied to the mid-sternal area and manipulated to locate the parasternal location that yields optimal heart tones. The IMSR is then placed on top of the precordial Doppler probe in the sulcus of the inter-mammary cleft, positioned such that the long axis spans the sternum from the manubrium to the costal margin near the xiphoid process (Figure 2).

Finally, the tissues of the two mammary complexes are manipulated anteriorly, relative to the sternum, and then medially until they adhere to the IMSR. Care is taken not to allow the adhesive surface of the IMSR to come in contact with the areola of the nipple because of the delicate nature of that surface.

## Discussion

The risk of a VAE is increased by an operative positioning that elevates the head above the heart because this can establish an intravenous pressure gradient that is subatmospheric [5]. Although not as sensitive as transesophageal echocardiography, the precordial Doppler ultrasonic probe is nonetheless extremely sensitive in the detection of VAE and at the same time is an inexpensive and very non-invasive modality. Historically, the primary limitations of precordial Doppler monitoring for VAE have been obesity and difficulty maintaining probe positioning against gravitational forces that favor separation of the probe from the precordium. The IMSR has proven very effective at circumventing those two difficulties.

Since the development of the IMSR by the senior author, J.C.D., it has been successfully utilized at our institution in innumerable neurosurgical procedures that require lateral positioning, and on occasion in obese patients in the sitting position. The noninvasive quality of both the precordial Doppler and the IMSR has not conferred any additional risk to the patients and has remained without associated complications. Future studies may investigate doppler measurements with and without IMSR to further assess efficacy.

## Conclusions

The IMSR is a safe and effective method for securing the precordial Doppler probe in close apposition to the chest wall during procedures that require the use of lateral positioning.
